# A highly efficient nano-Fe_3_O_4_ encapsulated-silica particles bearing sulfonic acid groups as a solid acid catalyst for synthesis of 1,8-dioxo-octahydroxanthene derivatives

**DOI:** 10.1007/s11051-013-2026-2

**Published:** 2013-10-09

**Authors:** Hossein Naeimi, Zahra Sadat Nazifi

**Affiliations:** Department of Organic Chemistry, Faculty of Chemistry, University of Kashan, 87317 Kashan, Islamic Republic of Iran

**Keywords:** Magnetic nanoparticle, Silica, 1,8‐Dioxo-octahydroxanthene, Dimedone, Aromatic aldehydes, Nanostructured catalyst

## Abstract

**Abstract:**

The functionalization of silica-coated Fe_3_O_4_ magnetic nanoparticles (Fe_3_O_4_@SiO_2_) using chlorosulfonic acid were afforded sulfonic acid-functionalized magnetic Fe_3_O_4_ nanoparticles (Fe_3_O_4_@SiO_2_–SO_3_H) that can be applied as an organic–inorganic hybrid heterogeneous catalyst. The used Fe_3_O_4_ magnetic nanoparticles are 18–30 nm sized that was rapidly functionalized and can be used as catalyst in organic synthesis. The prepared nanoparticles were characterized by X-ray diffraction analysis, magnetization curve, scanning electron microscope, dynamic laser scattering, and FT-IR measurements. The resulting immobilized catalysts have been successfully used in the synthesis of 1,8‐dioxo-octahydroxanthene derivatives under solvent free condition. This procedure has many advantages such as; a much milder method, a shorter reaction time, a wide range of functional group tolerance, and absence of any tedious workup or purification. Other remarkable features include the catalyst can be reused at least five times without any obvious change in its catalytic activity. This procedure also avoids hazardous reagents/solvents, and thus can be an eco-friendly alternative to the existing methods.

**Graphical Abstract:**

A highly efficient nano-Fe_3_O_4_ encapsulated-silica particles bearing sulfonic acid groups as a solid acid catalyst for synthesis of 1,8-dioxo-octahydroxanthene derivatives
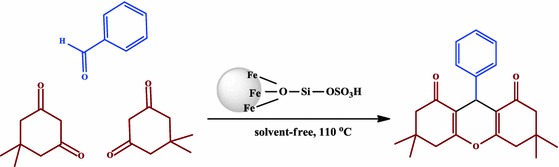

**Electronic supplementary material:**

The online version of this article (doi:10.1007/s11051-013-2026-2) contains supplementary material, which is available to authorized users.

## Introduction

Recently, Fe_3_O_4_ magnetic nanoparticles (MNPs) have been intensively investigated because of their superparamagnety, high coercively, and low Curie temperature (Kim and Kim 2003; Raj and Moskowitz 2002; Beydoun et al. 2000; McMichael et al. 1992). In addition to these characters, MNPs are also non-toxic and biocompatible.

MNPs have been used in various fields such as sealing, oscillation damping, information storage, and electronic devices (Lee et al. 2002; Yang et al. 2005; Caruntu et al. 2004; Zhang et al. 2004). One of the rapidly developing applications of MNPs in recent years is in biomedical areas, including rapid biologic separation and drug delivery (Tan et al. 2005; Garcia et al. 2007; Wang et al. 2005; Liao and Chen 2002). However, unmodified MNPs tend to aggregate because of their high specific area and strong inter particle interaction, which limit their utilization. Therefore, it is necessary to develop strategies for the chemical stabilization of the naked MNPs against aggregation over a long period. Although the development of more efficient and versatile approaches to functionalized MNPs is very important. Therefore, the outer shell of silica not only protects the inner magnetite core from oxidation but also provides sites for surface functionalization with chlorosulfonic acid. Despite the formation of Fe_3_O_4_@SiO_2_–SO_3_H materials not only stabilized the MNPs, but also endowed the MNPs with functionality (Iaoying Yang et al. 2010).

Xanthenes and their derivatives are an important class of heterocyclic compounds, which are widely used in biological applications, such as antibacterial activities (Karthikeyana and Pandurangan 2009), anti-inflammatory (Poupelin et al. 1978), and antiviral properties (Hajipour et al. 2010). Furthermore, some of the heterocycles based on xanthenes have found application as photodynamic therapy for destroying the tumor cells (Ion et al. 1998). The other useful applications of these heterocycles can be used as dyes (Imani Shakibaei et al. 2007), laser technology (Ahmad et al. 2002), and in fluorescent materials for visualization of biomolecules (Knight and Stephens 1989).

In this context, we were prepared Fe_3_O_4_ nanoparticles by chemical co-precipitation method and subsequently coated with tetraethoxysilane (TEOS) via silanization reaction. Grafting of chlorosulfonic acid on the Fe_3_O_4_@SiO_2_ nanoparticles was afforded sulfamic acid-functionalized MNPs (Fe_3_O_4_@SiO_2_–SO_3_H). They were found to be a mild and efficient solid acid nano catalyst for the one-pot synthesis of 1,8‐dioxo-octahydroxanthenes under solvent-free conditions.

In this method, the corresponding 1,8‐dioxo-octahydroxanthenes were afforded in shorter reaction times and excellent yields with high purity. This green procedure has many obvious advantages compared to those reported in the previous literatures, including avoiding the use of harmful catalysts, easy workup of the reaction, excellent yields, short routine, and simplicity of the methodology.

## Experimental section

### Materials

All commercially available reagents were used without further purification and purchased from the Merck Chemical Company in high purity. The used solvents were purified by standard procedure.

### Apparatus

FT-IR spectra were obtained as KBr pellets on a Perkin-Elmer 781 spectrophotometer and on an Impact 400 Nicolet FT-IR spectrophotometer. ^1^H NMR and ^13^C NMR were recorded in CDCl_3_ solvents on a Bruker DRX-400 spectrometer with tetramethylsilane as internal reference. Nanostructures were characterized using a Holland Philips Xpert X-ray powder diffraction (XRD) diffractometer (CuK, radiation, *k* = 0.154056 nm), at a scanning speed of 2°/min from 10° to 100° (2θ). Thermo gravimetric analyses (TGA) were conducted on a Rheometric Scientific Inc. 1998 thermal analysis apparatus under a N_2_ atmosphere at a heating rate of 10 °C/min. Scanning electron microscope (SEM) of nanoparticles was performed on a FESEM Hitachi S4160. Dynamic laser scattering (DLS) was performed on Malvern ZEN 3600. The Bandelin ultrasonic HD 3200 with probe model KE 76.6 mm diameter, was used to produce ultrasonic irradiation and homogenizing the reaction mixture. Melting points obtained with a Yanagimoto micro melting point apparatus are uncorrected. The purity determination of the substrates and reaction monitoring were accomplished by TLC on silica-gel polygram SILG/UV 254 plates (from Merck Company).

## Preparation of catalyst

### Preparation of nano-Fe_3_O_4_

Fe_3_O_4_ MNPs were prepared according to a previously reported procedure by the chemical co-precipitation method (Hu et al. 2012). Typically, FeCl_3_·6H_2_O (2.7 g) and FeCl_2_·4H_2_O (1 g) were dissolved in 100 ml of 1.2 mmol l^−1^ aqueous HCl by ultrasonic bath for 30 min. Then, 1.25 mol l^−1^ aqueous NaOH (150 ml) was added under vigorous stirring and a black precipitate was immediately formed. The resulting transparent solution was heated at 80 °C with rapid mechanical stirring under N_2_ atmosphere. After vigorous stirring for 2 h, the precipitate was magnetically separated and washed thoroughly with water until the supernatant liquor reached neutrality (pH ~7).

### Preparation of nano-Fe_3_O_4_@SiO_2_

This precursor was prepared according to the reported method (Yang et al. 2009). MNPs (1 g) were initially diluted via the sequential addition of water (20 ml), ethanol (60 ml), and concentrated aqueous ammonia (2 ml, 25 wt%). The resulting dispersion was then homogenized by ultrasonic. A solution of TEOS (0.5 ml) in ethanol (10 ml) was then added to the dispersion in a drop-wise manner under continuous mechanical stirring. After vigorous stirring for 16 h, the product were collected by an external magnetic and washed three times with ethanol. Finally, the products were dried under vacuum at 70 °C for 5 h.

### Preparation of Fe_3_O_4_@SiO_2_–SO_3_H

Fe_3_O_4_@SiO_2_–SO_3_H microspheres were synthesized as following; firstly, 1 g of Fe_3_O_4_@SiO_2_ microspheres was dispersed in dry CH_2_Cl_2_ (10 ml) by ultrasonic bath for 30 min. Subsequently, chlorosulfonic acid (1 ml) was added drop-wise to a cooled (ice-bath) solution of Fe_3_O_4_@SiO_2_ (1 g) over a period of 30 min at room temperature. After completion of the addition, the mixture was stirred for a further 6 h until to allow for the complete dissipation of HCl from the reaction vessel. The resulted MNPs were separated using an external magnet and washed with ethanol and water before being dried in an oven at 70 °C to give Fe_3_O_4_@SiO_2_–SO_3_H as a brown powder.

### General procedure for the synthesis of 1,8-dioxo-octahydroxanthenes

A mixture of an aromatic aldehydes (1 mmol), dimedone (2 mmol) and nano Fe_3_O_4_@SiO_2_–SO_3_H (0.05 g) was heated at 110 °C under solvent-free conditions. The progress of the reactions was monitored by TLC (ethyl acetate/petroleum ether 3/7). After completion of the reaction, the reaction mixture was cooled and CH_2_Cl_2_ (5 ml) was added and the catalyst was separated by an external magnet and reused for the next experiment. The reaction mixture was concentrated on a rotary evaporator under reduced pressure. The residue was purified by recrystallization from ethanol. They were characterized by comparison of their physical and spectral data with those of authentic samples (Girijesh et al. 2011; Mahdavinia et al. 2009; Swapna et al. 2011; Kantevari et al. 2007).

*9*-*Phenyl*-*3,3,6,6*-*tetramethyl*-*1,2,3,4,5,6,7,8*-*octahydroxanthene*-*1,8*-*dione (3a):* white solid, m.p. = 202–203 °C, (m.p. = 203–204 °C Girijesh et al. 2011), IR (KBr)/υ(cm^−1^): 2956, 1666, 1460, 1363, 1198, 698; ^1^H NMR (CDCl_3,_ 400 MHz)/δ ppm: 7.27–7.30 (t, 3H, *J* = 7.6 Hz, ArH), 7.20–7.23 (t, 3H, *J* = 7.2 Hz, ArH), 7.10–7.12 (t, 1H, *J* = 7.2 Hz, ArH), 4.75 (s, 1H, CH); 2.47 (s, 4H, 2CH_2_), 2.15–2.26 (q, 4H, 2CH_2_), 1.10 (s, 6H, 2CH_3_), 0.99 (s, 6H, CH_3_).

*9*-*(4*-*Nitrophenyl)*-*3,3,6,6*-*tetramethyl*-*1,2,3,4,5,6,7,8*-*octahydroxanthene*-*1,8*-*dione (3g):* pale yellow solid, m.p. = 224–226 °C, (m.p. = 226–227 °C, Mahdavinia et al. 2009), IR (KBr)/υ(cm^−1^): 2959, 1664, 1517, 1359, 1199, 865; ^1^H NMR (CDCl_3,_ 400 MHz)/δ ppm: 8.08–8.10 (d, 2H, *J* = 8.0 Hz, ArH), 7.46–7.48 (d, 2H, *J* = 8.0 Hz, ArH), 4.48 (s, 1H, CH), 2.50 (s, 4H, 2CH_2_), 2.14–2.28 (q, 4H, *J* = 16.4 Hz, 2CH_2_), 1.12 (s, 6H, 2CH_3_), 0.99 (s, 6H, CH_3_); ^13^C NMR (CDCl_3,_ 100 MHz)/δ ppm: 196.32, 169.32, 163.02, 151.58, 146.45, 129.38, 123.42, 114.49, 50.60, 40.82, 32.38, 32.24, 29.24, 27.27.

*9*-*(3*-*Nitrophenyl)*-*3,3,6,6*-*tetramethyl*-*1,2,3,4,5,6,7,8*-*octahydroxanthene*-*1,8*-*dione (3f):* white solid, m.p. = 171–172 °C, (m.p. = 168–170 °C, Mahdavinia et al. 2009), IR (KBr)/υ(cm^−1^): 2960, 1664, 1527, 1357, 1198, 1138, 814; ^1^H NMR (CDCl_3,_ 400 MHz)/δ ppm: 7.39–8.01 (m, 4H, ArH), 4.84 (s, 1H, CH), 2.51 (s, 4H, 2CH_2_), 2.15–2.28 (q, 4H, *J* = 16.4 Hz, 2CH_2_), 1.12 (s, 6H, 2CH_3_), 0.99 (s, 6H, CH_3_); ^13^C NMR/(CDCl_3,_ 100 MHz)/δ ppm: 196.36, 169.03, 148.33, 146.31, 135.37, 128.80, 122.55, 121.66, 114.55, 50.64, 40.82, 32.25, 32.10, 29.21, 27.31.

*9*-*(4*-*Chlorophenyl)*-*3,3,6,6*-*tetramethyl*-*1,2,3,4,5,6,7,8*-*octahydroxanthene*-*1,8*-*dione (3c):* white solid, m.p. = 231–233 °C, (m.p. = 230–232 °C, Swapna et al. 2011), IR (KBr)/υ(cm^−1^): 2956, 1663, 1469, 1362, 1197, 1139, 846; ^1^H NMR (CDCl_3,_ 400 MHz)/δ ppm: 7.27–7.52 (d, 2H ArH), 4.84 (s, 1H, CH), 2.51 (s, 4H, 2CH_2_), 2.15–2.28 (q, 4H, *J* = 16.4 Hz, 2CH_2_), 1.12 (s, 6H, 2CH_3_), 0.99 (s, 6H, CH_3_).

*9*-*(4*-*Chloro*-*3*-*nitrophenyl)*-*3,3,6,6*-*tetramethyl*-*1,2,3,4,5,6,7,8*-*octahydroxanthene*-*1,8*-*dione (3h):* white solid, m.p. = 251–253 °C, IR (KBr)/υ(cm^−1^): IR (KBr)/υ(cm^−1^): 2961, 1665, 1534, 1361, 1198, 828; ^1^H NMR (CDCl_3,_ 400 MHz)/δ ppm: 7.67 (s, 1H, ArH), 7.63–7.66 (d, 1H, *J* = 8.4 Hz, ArH); 7.40–7.42 (d, 1H, *J* = 8.4 Hz, ArH), 4.77 (s, 1H, CH); 2.50 (s, 4H, 2CH_2_), 2.17–2.28 (q, 4H, *J* = 16.4 Hz, 2CH_2_), 1.12 (s, 6H, 2CH_3_), 1.02 (s, 6H, CH_3_).

*9*-*(2,4*-*Dichlorophenyl)*-*3,3,6,6*-*tetramethyl*-*1,2,3,4,5,6,7,8*-*octahydroxanthene*-*1,8*-*dione (3d):* white solid, m.p. = 250–252 °C, (m.p. = 248–250 °C, Mahdavinia et al. 2009), IR (KBr)/υ(cm^−1^): 2943, 2930, 1717, 1657, 1587, 1383, 1169; ^1^H NMR (CDCl_3,_ 400 MHz)/δ ppm: 7.37 (s, 1H, ArH), 7.25–7.27 (d, 1H, *J* = 8.0 Hz, ArH), 7.14–7.16 (d, 1H, *J* = 8.4 Hz, ArH), 4.95 (s, 1H, CH), 2.45 (s, 4H, 2CH_2_), 2.14–2.25 (q, 4H, *J* = 16.0 Hz, 2CH_2_), 1.11 (s, 6H, 2CH_3_), 1.02 (s, 6H, CH_3_).

*9*-*(3*-*Methoxy)*-*3,3,6,6*-*tetramethyl*-*1,2,3,4,5,6,7,8*-*octahydroxanthene*-*1,8*-*dione (3l):* white solid, m.p. = 181–182 °C, (m.p. = 179–181 °C, Mahdavinia et al. 2009), IR (KBr)/υ(cm^−1^): 2959, 1662, 1485, 1363, 1274, 1201, 1048, 763; ^1^H NMR (CDCl_3,_ 400 MHz)/δ ppm: 7.15–7.18 (t, 1H, *J* = 8.0 Hz, ArH), 6.86–6.89 (d, 1H, ArH), 6.64–6.67 (d, 1H, ArH), 4.74 (s, 1H, CH), 2.46 (s, 4H, 2CH_2_), 2.16–2.26 (q, 4H, *J* = 16.4 Hz, 2CH_2_), 1.10 (s, 6H, 2CH_3_), 1.01 (s, 6H, CH_3_).

*9*-*(4*-*Methoxy)*-*3,3,6,6*-*tetramethyl*-*1,2,3,4,5,6,7,8*-*octahydroxanthene*-*1,8*-*dione (3m):* white solid, mp: 245–247 °C, (m.p. = 243–245 °C, Mahdavinia et al. 2009), IR (KBr)/υ(cm^−1^): 2957, 1667, 1510, 1461, 1359, 1260, 1194, 1137, 1032, 568; ^1^H NMR (CDCl_3,_ 400 MHz)/δ ppm: 7.19–7.21 (d, 2H, *J* = 8.4 Hz, ArH), 6.74–6.79 (d, 2H, ArH), 6.64–6.67 (d, 1H, ArH), 4.70 (s, 1H, CH), 3.73 (s, 3H, OCH_3_), 2.46 (s, 4H, 2CH_2_), 2.14–2.25 (q, 4H, *J* = 16.4 Hz, 2CH_2_), 1.10 (s, 6H, 2CH_3_), 0.99 (s, 6H, CH_3_); ^13^C NMR/(CDCl_3,_ 100 MHz)/δ ppm: 196.47, 162.08, 157.96, 136.51, 131.99, 129.31, 115.79, 114.33, 113.46, 55.10, 50.78, 40.86, 32.19, 30.97, 29.28, 27.34, 23.43.

## Results and discussion

### Characterization of Fe_3_O_4_@SiO_2_–SO_3_H as solid acid catalyst

**Scheme 1:**
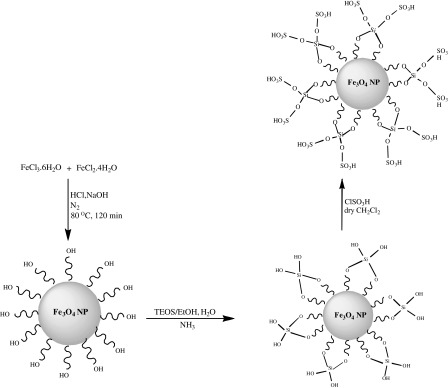
Preparation steps for fabricating sulfamic acid-functionalized magnetic Fe_3_O_4_ nanoparticles

The MNPs of 18–22 nm were prepared by co-precipitation via iron(II) and iron(III) ions. For the surface modification, the MNPs coated with a layer of silica using the (TEOS) by co-precipitation method (Yang et al. 2009) to provide reaction sites for further functionalization and thermal stability. Ultimately, the reaction of Fe_3_O_4_@SiO_2_ with chlorosulfonic acid led to sulfamic acid-functionalized magnetic Fe_3_O_4_ nanoparticles (Fe_3_O_4_@SiO_2_–SO_3_H) (Scheme 1). The pH measurement of Fe_3_O_4_@SiO_2_–SO_3_H (10 % w/v) was obtained about 1.36.

**Fig. 1:**
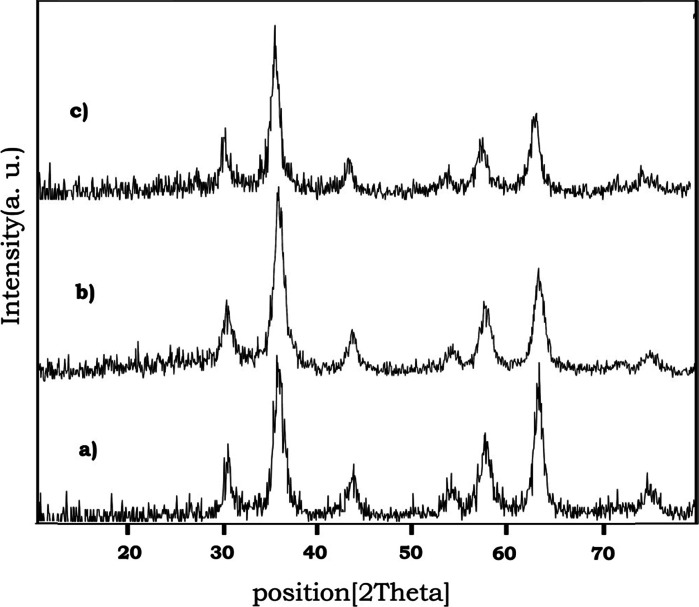
XRD patterns of *a* nano Fe_3_O_4_ *b* Fe_3_O_4_@SiO_2_ *c* Fe_3_O_4_@SiO_2_–SO_3_H

The X-ray diffraction patterns of Fe_3_O_4_, Fe_3_O_4_@SiO_2_ and Fe_3_O_4_@SiO_2_–SO_3_H are shown in Fig. 1. The position and relative intensities of all peaks confirm well with standard XRD pattern of Fe_3_O_4_ indicating retention of the crystalline cubic spinel structure during functionalization of MNPs. Characteristic peak of SiO_2_ in core shell structure has been hidden under weak peak of Fe_3_O_4_ at 2θ = 30. The average MNPs core diameter was calculated to be 22 nm from the XRD results by Scherrer’s equation, *D* = *kλ*/*β*cos*θ* where k is a constant (generally considered as 0.94), *λ* is the wavelength of Cu Ka (1.54 Å), *β* is the corrected diffraction line full-width at half-maximum (FWHM), and *θ* is Bragg’s angle (Massart 1981).

**Fig. 2:**
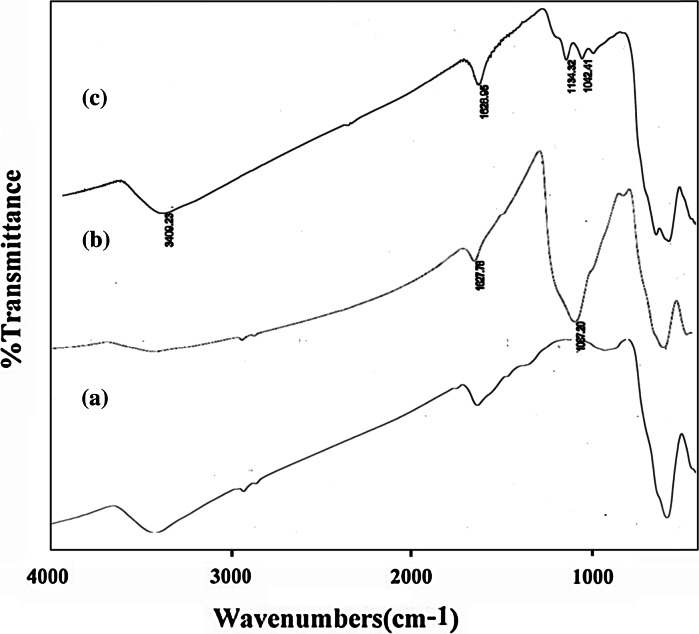
The comparative FT-IR spectra of *a* nano Fe_3_O_4_ *b* Fe_3_O_4_@SiO_2_ *c* Fe_3_O_4_@SiO_2_–SO_3_H

The FT-IR spectra of catalyst are presented the band in the region of 572 cm^−1^ is attributed to the stretching vibrations of the (Fe–O) bond and the band at about 1,100 cm^−1^ belongs to (Si–O) stretching vibrations. FT-IR analysis was used to characterize the presence of the –SO_3_H groups on the surface of the MNPs (Fig. 2). As shown in Fig. 2 **c**, the FT-IR spectra of Fe_3_O_4_@SiO_2_–SO_3_H was clearly different from those of Fe_3_O_4_ (Fig. 2a) and Fe_3_O_4_@SiO_2_ (Fig. 2b). For sulfonic acid functional group, the appeared peaks in 1,042 and 1,134 cm^−1^ are related to the stretching of the S–O bonds. A peak appeared at about 3,409 cm^−1^ due to the stretching of OH groups in the SO_3_H (Fig. 2c).

**Fig. 3:**
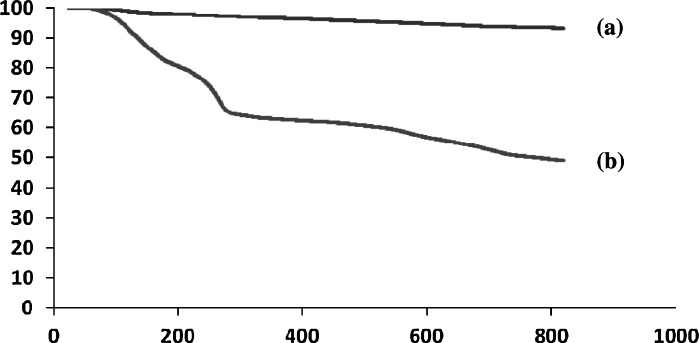
TGA curve of *a* Fe_3_O_4_@SiO_2_ *b* Fe_3_O_4_@SiO_2_–SO_3_H

A thermogravimetric analysis (TGA) was used to study the thermal stability of the acid catalyst (Fig. 3). The TGA curve was divided into several regions corresponding to different mass lose ranges. The first region, which occurred below 150 °C, displayed a mass loss that was attributable to the loss of adsorbed solvent or trapped water from the catalyst. A weight loss of approximately 10 % weight occurred between 150 and 500 °C that was likely a consequence of the loss of SO_3_H groups. The occurrence of further mass losses at higher temperature was resulted from the decomposition of silica shell (Nemati et al. 2012). Thus; the catalyst was stable up to 250 °C, confirming that it could be safely used in organic reactions at temperatures between the ranges of 80–150 °C.

**Fig. 4:**
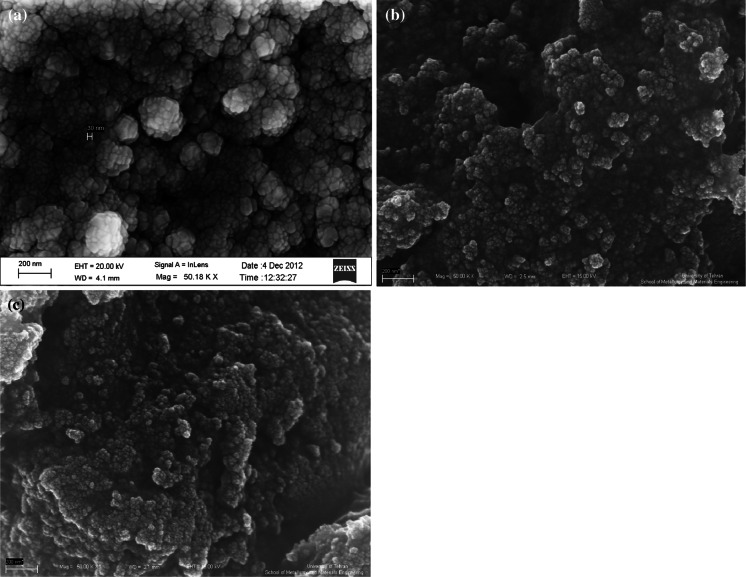
The SEM image of **a** Fe_3_O_4_, **b** Fe_3_O_4_@SiO_2_, **c** Fe_3_O_4_@SiO_2_–SO_3_H

The SEM image was shown that MNPs have a mean diameter of about 20 nm and a nearly spherical shape in Fig. 4a, b shows that Fe_3_O_4_@SiO_2_ nanoparticles still keep the morphological properties of Fe_3_O_4_ except for a slightly larger particle size and smoother surface, which silica are uniform coated on the Fe_3_O_4_ particles to form silica shell in compared to the Fe_3_O_4_@SiO_2_. The SEM image shown in Fig. 4 **c** demonstrates that Fe_3_O_4_@SiO_2_–SO_3_H nanoparticles are nearly spherical with more than 20 nm in size.

**Fig. 5:**
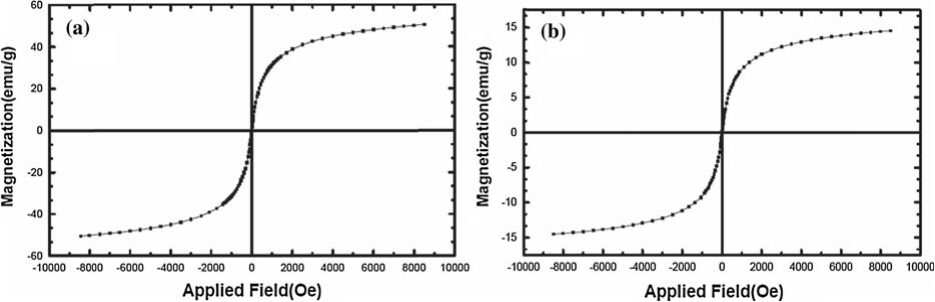
Magnetization curves for the prepared **a** Fe_3_O_4_@SiO_2_ and **b** Fe_3_O_4_@SiO_2_–SO_3_H at 40 °C

The magnetization curve for Fe_3_O_4_@SiO_2_ nanoparticles and Fe_3_O_4_@SiO_2_–SO_3_H are shown in Fig. 5. Room temperature specific magnetization (*M*) versus applied magnetic field (*H*) curve measurements of the sample indicate a saturation magnetization value (Ms) of 15 emu g^−1^, lower than that of bare MNPs (50.86 emu g^−1^) due to the coated shell.

**Fig. 6:**
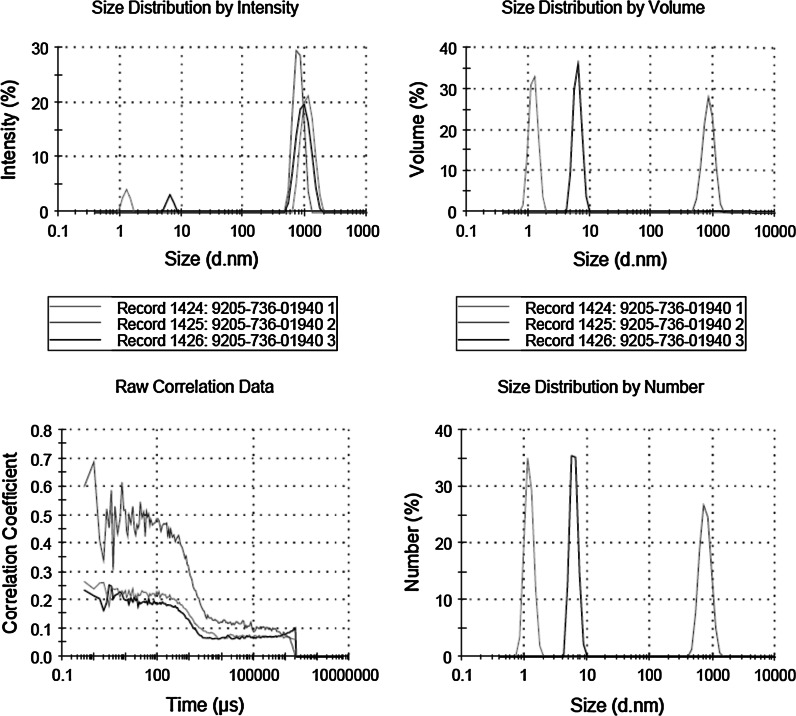
The dynamic laser scattering (DLS) measurement of nano Fe_3_O_4_@SiO_2_–SO_3_H

The dynamic laser scattering (DLS) measurement of Fe_3_O_4_@SiO_2_–SO_3_H nanoparticles was shown in Fig. 6. In order to determine the fraction of the particle population that aggregates, comparisons between the intensity averaged DLS data and number averaged DLS data were made. From this slurry, an aqueous stock dispersion (100 ml acetone at 5 g Fe_3_O_4_@SiO_2_–SO_3_H) was prepared using an ultrasonic bath for 30 min.

**Fig. 7:**
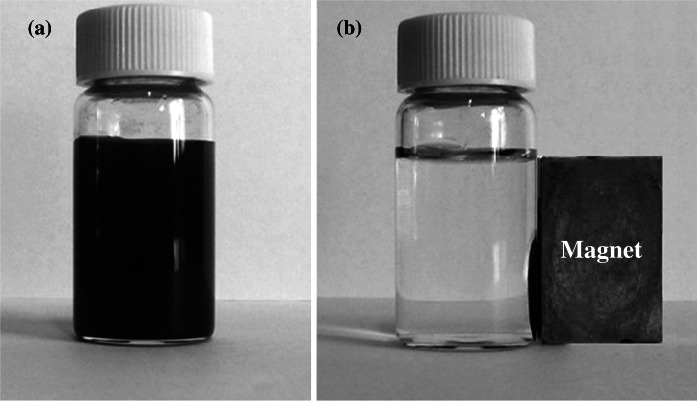
Digital camera images of the aqueous solution **a** with dispersed magnetic Fe_3_O_4_@SiO_2_–SO_3_H composite particles and **b** after applied magnetic field

Figure 7a shows the photograph of Fe_3_O_4_@SiO_2_–SO_3_H microspheres that dispersed in water. After a magnet was placed aside, the black microspheres can be magnetized in 5 min, leaving a clear solution (Fig. 7b). That is to say, the Fe_3_O_4_@SiO_2_–SO_3_H nanoparticles were shown good magnetic responsibility even if the SiO_2_–SO_3_H layer was increased to 20 nm.

### Evaluation of the catalytic activity of Fe_3_O_4_@SiO_2_–SO_3_H in the synthesis 1,8-dioxo-octahydro-xanthenes

In this research, a simple energy, eco-friendly and convenient method for the synthesis of 1,8‐dioxo-octahydroxanthenes using Fe_3_O_4_@SiO_2_–SO_3_H as new catalyst are described. Initially, in order to optimize the reaction conditions, it is considered to represent the reaction of dimedone and benzaldehyde in a 2:1 ratio to afford the xanthene **3a** under various reaction conditions was performed for an appropriate time (Table 1).

The obtained results from the reaction to determine the optimum amount of catalyst are presented in Table 1. As can be seen from this Table, the best results were obtained using 0.05 g of catalyst in the reaction of benzaldehyde (1 mol) with dimedone (2 mol) (Table 1, entry 5).

**Table 1:** The synthesis of (**3a**) under a different amount of catalyst

Entry	Catalyst loading (g)	Time (min)	Yield^a^ (%)
1	–	30	0
2	0.02	15	45
3	0.03	15	55
4	0.04	15	75
5	0.05	4	97
6	0.06	4	97

^a^Yields of isolated pure product

**Scheme 2:**
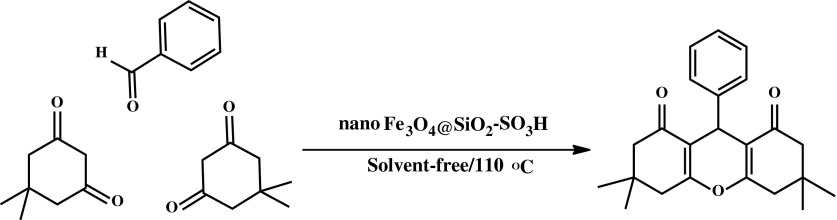
Synthesis of 1,8-dioxo-octahydroxanthene under solvent-free conditions

After optimization of the reaction conditions, the reaction of dimedone with various aldehydes was carried out in according to the general experimental procedure (Scheme 2). In all the cases, the corresponding xanthenediones were obtained in high to excellent yields and short reaction times. The obtained similar products are summarized in Table 2.

**Table 2:** Synthesis of 1,8‐dioxo-octahydroxanthenes (**3a**–**m**) catalyzed by Fe_3_O_4_@SiO_2_–SO_3_H under solvent-free conditions at 110 °C

Entry	Aldehyde (R**)**	Product	Time (min)	Yield	M.p. (°C)
1	C_6_H_5_	**3a**	4	97	202–203
2	*o*-Cl·C_6_H_4_	**3b**	8	90	225–226
3	*p*-Cl·C_6_H_4_	**3c**	5	95	231–233
4	2,4-Cl_2_·C_6_H_3_	**3d**	7	92	250–252
5	*o*-NO_2_·C_6_H_4_	**3e**	8	89	256–258
6	*m*-NO_2_·C_6_H_4_	**3f**	5	90	171–172
7	*p*-NO_2_·C_6_H_4_	**3g**	4	96	224–226
8	4-Cl-3-NO_2_·C_6_H_3_	**3h**	6	94	251–253
9	*o*-OH·C_6_H_4_	**3i**	8	88	231–233
10	*p*-OH·C_6_H_4_	**3j**	4	93	247–248
11	*p*-Me·C_6_H_4_	**3k**	5	95	223–224
12	*m*-OMe·C_6_H_4_	**3l**	6	93	181–182
13	*p*-OMe·C_6_H_4_	**3m**	4	95	245–247

^a^Reaction condition: aldehyde (1 mmol), dimidone (2 mmol), Fe_3_O_4_@SiO_2_–SO_3_H (0.05 g)

^b^Yields of isolated pure product

The presence of electron-donating (alkoxy or hydroxyl group) or electron-withdrawing groups (nitro or halide group) on the aromatic ring of the aldehydes did not have much effect on the reaction such that to afford respective products with high yields. While *para*-substituted aldehydes were given good results in compared to the *ortho*-substituents. There is more steric hindrance for the ortho substituted aldehydes (*o*-OCH_3_, –OH, –Cl, –NO_2_) on the product formation than the *para*-substituted (*p*-OCH_3_, –OH, –Cl, –NO_2_) aldehydes.

**Fig. 8:**
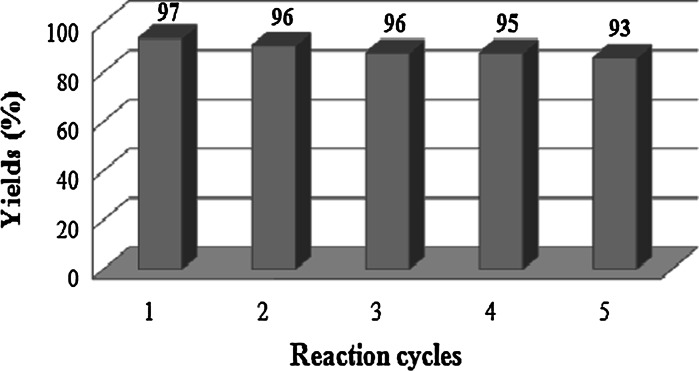
Reusability of Fe_3_O_4_@SiO_2_–SO_3_H in the reaction of dimedone, 4-nitrobenzaldehyde under solvent-free conditions

The possibility of recycling the catalyst was examined through the reaction of dimedone and 4-nitrobenzaldehyde catalyzed by Fe_3_O_4_@SiO_2_–SO_3_H nanoparticles under optimized conditions. Upon completion of the reaction, the catalyst was separated by an external magnet, washed with acetone, and the recycled catalyst was saved for the next reaction. The recycled catalyst could be reused five times without any decrease in catalytic activity so that the yields were ranged from 93 to 97 % (Fig. 8).

The structure of the obtained products was confirmed by IR, ^1^H NMR, and ^13^C NMR spectra. The infrared spectra of the **3g** exhibit a medium band at 2,959 cm^−1^ represents the presence of alkane protons (4CH_3_ groups). In addition, a band at 1,664 cm^−1^ represents the presence of general carbonyl groups (C=O stretching) and a strong band at 1,199 cm^−1^ confirms the presence of C–O bond stretching. In ^1^H NMR spectra of compound **3g**, the four methyl groups were appeared as two series of axial and equatorial methyl groups. Therefore, these protons were differently indicated as two singlet bands with six hydrogens at around 0.99 and 1.12 ppm, respectively. Due to the possibility of free rotation and conformational considerations, the four methyl groups are not becoming equivalent and appear as two singlet at different chemical shifts in all the compounds that is indicated the axial and equatorial positions. The proton at the bridge between two dimedone rings appears usually in the region 4.83 ppm and the signal around *δ* = 7.46–8.10 ppm is assigned to the protons of the aromatic rings (CH=CH). In ^13^C NMR, two carbon atoms of dimedone (CH_3_ groups) were symmetrical and give rise to one signal for each set of carbons. The carbon at the bridge between two dimedone rings is shown at 50.6 ppm. The carbonyl carbon atom appears in the expected region around 196.32 ppm.

### The proposed reaction mechanism

**Scheme 3:**
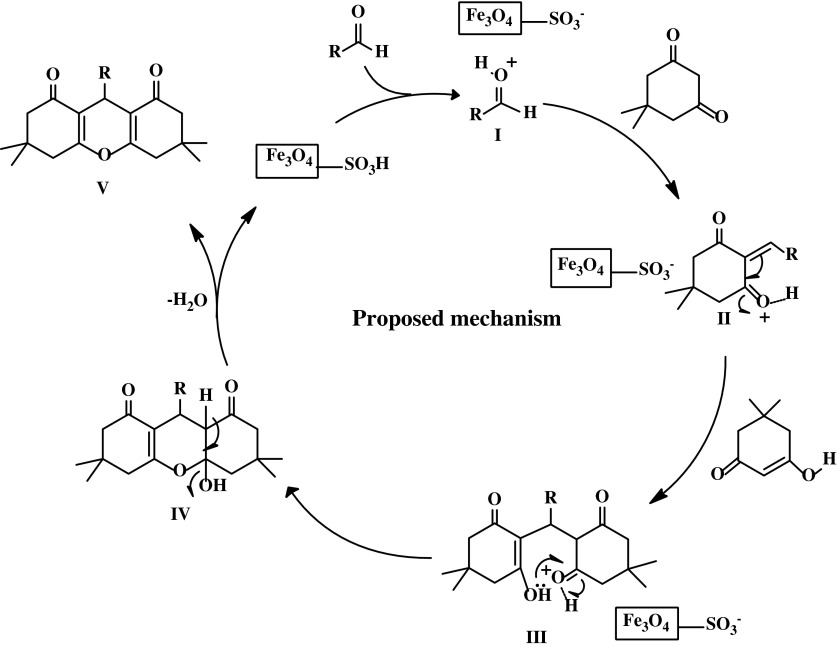
Proposed reaction mechanism

The formation of 1,8‐dioxo-octahydroxanthene from dimedone and aldehyde in the presence of Fe_3_O_4_@SiO_2_–SO_3_H as catalyst can be explained by a tentative mechanism is presented in Scheme 3. One molecule of dimedone (I) was firstly condensed with an activated aromatic aldehyde to provide intermediate II, which can be regarded as a fast Knoevenagel addition. Then the active methylene of the second molecule of dimedone reacted with intermediate II via conjugate Michael addition to produce the intermediate III, which undergoes intramolecular cyclodehydration to give the octahydroxanthene-1,8-dione (IV).

## Conclusion

In summary, we were described using Fe_3_O_4_@SiO_2_–SO_3_H as a reusable, readily available, inexpensive and efficient catalyst for the one-pot synthesis of 1,8‐dioxo-octahydroxanthenes. These compounds were prepared through treatment of dimedone with various aromatic aldehydes under solvent-free conditions at 110 °C. Our main strategy in this work is to develop a facile protocol, low cost, easily available catalyst, reduce reaction time, easy workup, and environmental friendliness.

## Electronic supplementary material

Below is the link to the electronic supplementary material.

Supplementary material 1 (PDF 842 kb)
