# Supplementary material for: A highly efficient nano-Fe3O4 encapsulated-silica particles bearing sulfonic acid groups as a solid acid catalyst for synthesis of 1,8-dioxo-octahydroxanthene derivatives
Source: J Nanopart Res. 2013 Oct 9;15(11):2026. doi: 10.1007/s11051-013-2026-2 (PMC3840292; doi:10.1007/s11051-013-2026-2)
Supplement: Supplementary file 1 — Supplementary material 1 (PDF 842 kb) [file 11051_2013_2026_MOESM1_ESM.pdf]

9-Phenyl-3,3,6,6-tetramethyl-1,2,3,4,5,6,7,8-octahydroxanthene-1,8-dione:

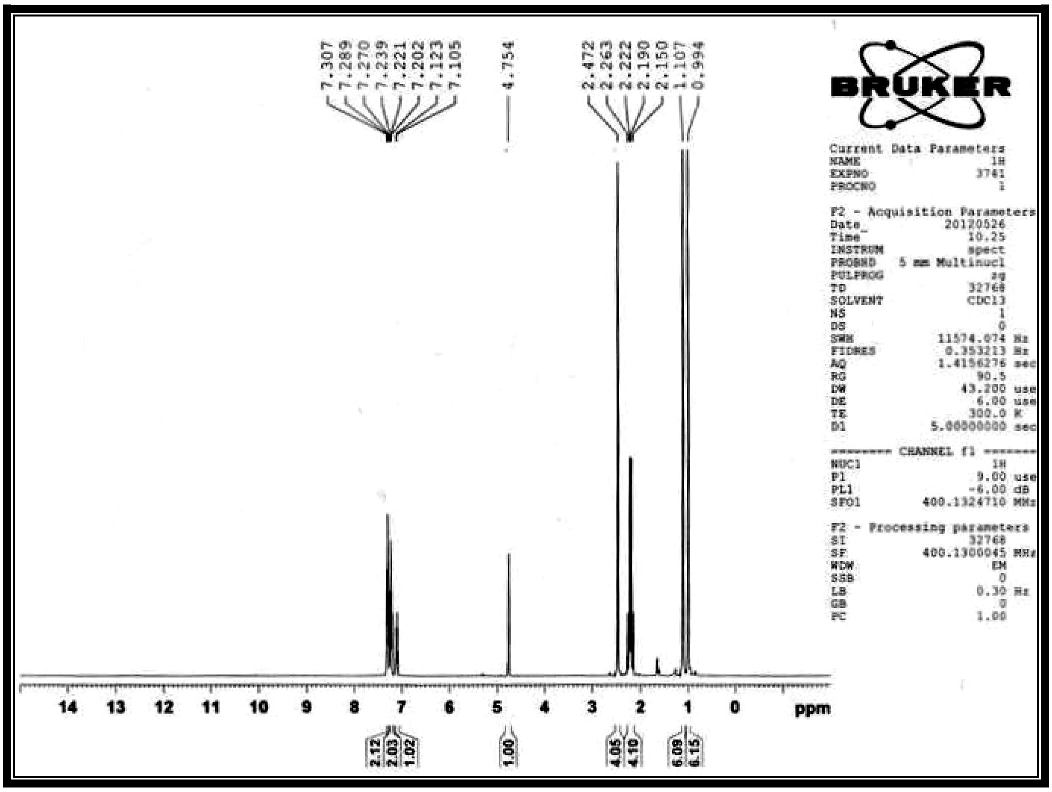

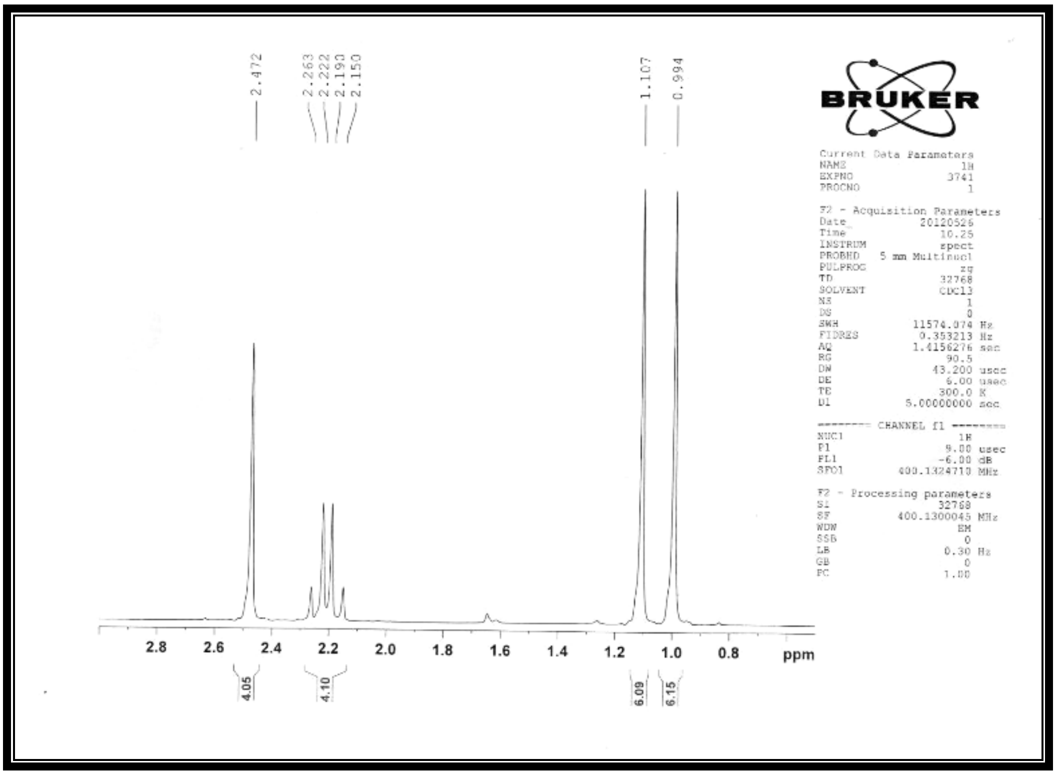

9-(4-Nitrophenyl)-3,3,6,6-tetramethyl-1,2,3,4,5,6,7,8-octahydroxanthene-1,8-dione:

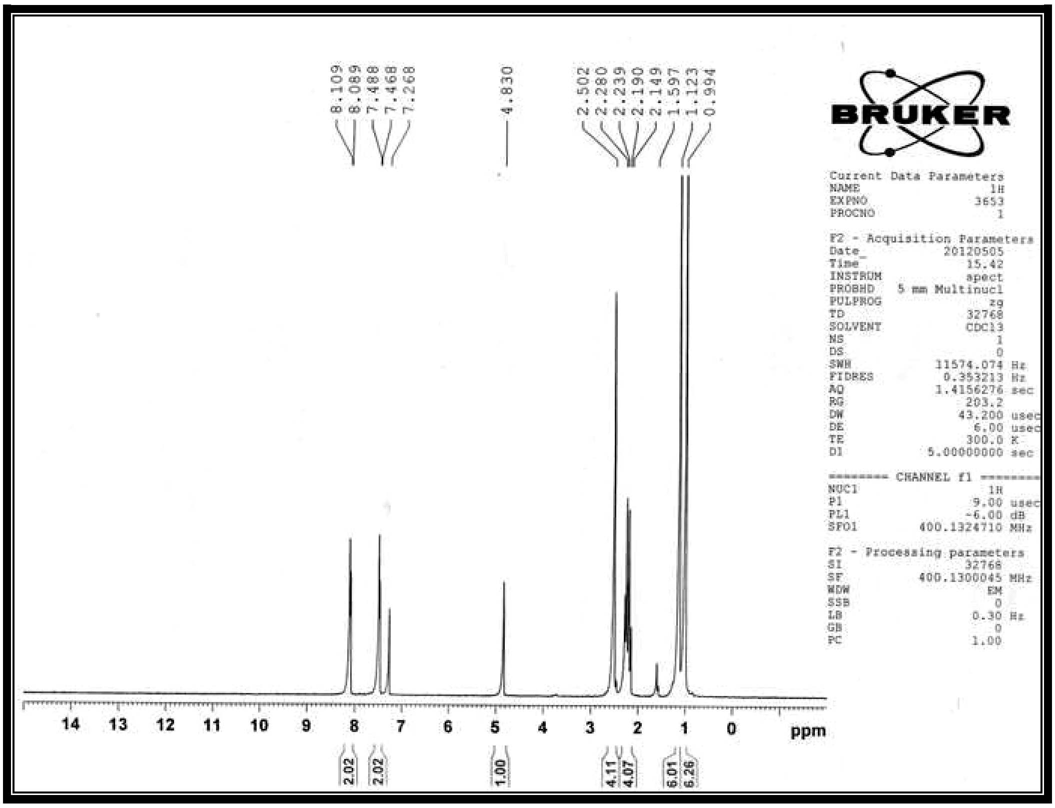

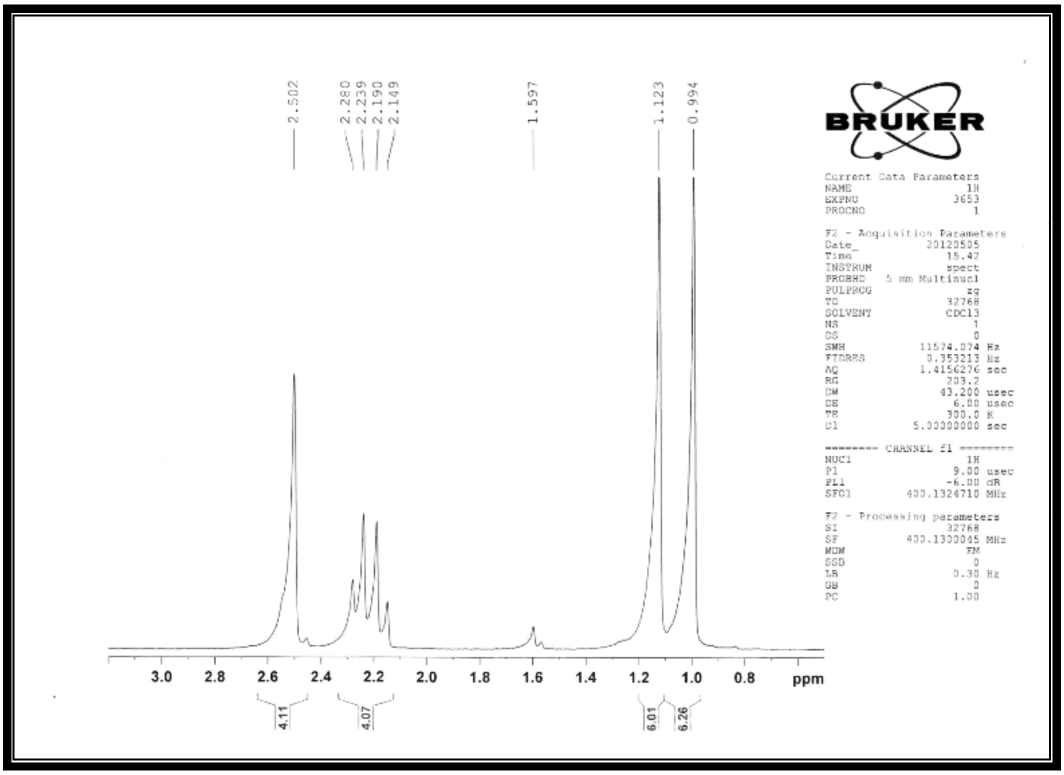

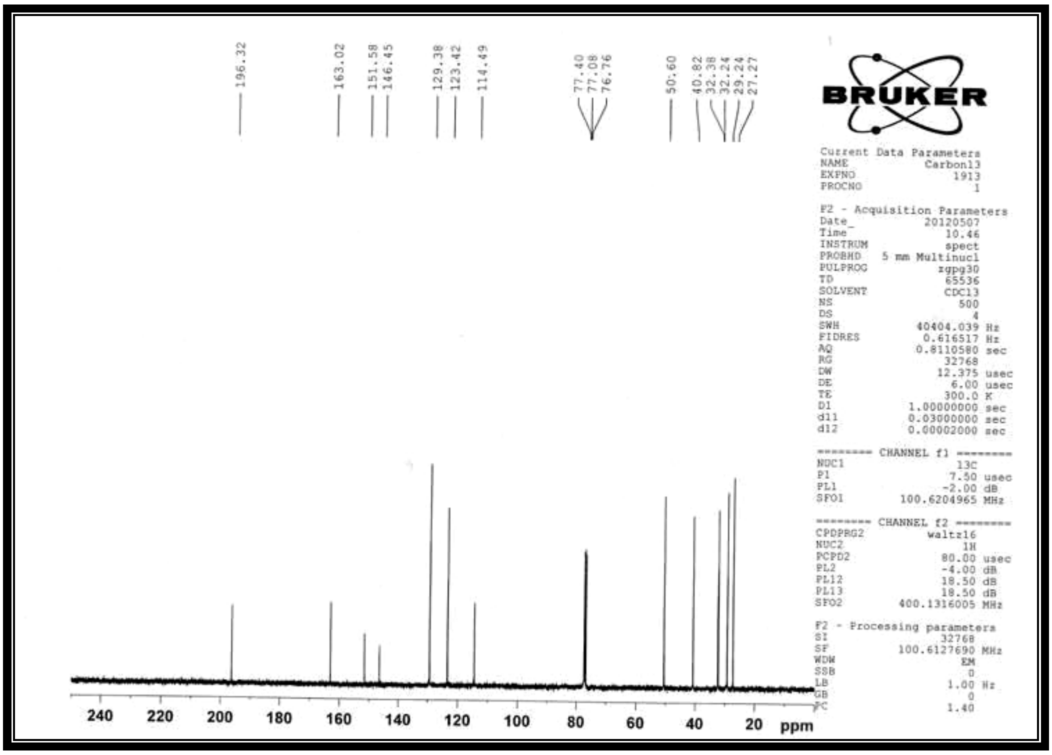
196.32
163.02
151.58
146.45
129.38
123.42
114.49
77.40
77.08
76.76
50.60
40.82
32.38
32.24
29.24
27.27
BRUKER
Current Data Parameters
NAME Carbon13
EXPNO 1913
PROCNO 1
F2 - Acquisition Parameters
Date_ 20120507
Time 10.46
INSTRUM spect
PROBHD 5 mm Multinucl
PULPROG zgpg30
TD 65536
SOLVENT CDCl3
NS 500
DS 4
SWH 40404.039 Hz
FIDRES 0.616517 Hz
AQ 0.8110580 sec
RG 32768
DW 12.375 usec
DE 6.00 usec
TE 300.0 K
D1 1.00000000 sec
d11 0.03000000 sec
d12 0.00002000 sec
======== CHANNEL f1 ========
NUC1 13C
P1 7.50 usec
PL1 -2.00 dB
SFO1 100.6204965 MHz
======== CHANNEL f2 ========
CPDPRG2 waltz16
NUC2 1H
PCPD2 80.00 usec
PL2 -4.00 dB
PL12 18.50 dB
PL13 18.50 dB
SFO2 400.1316005 MHz
F2 - Processing parameters
SI 32768
SF 100.6127690 MHz
WDW EM
SSB 0
LB 1.00 Hz
GB 0
PC 1.40
240 220 200 180 160 140 120 100 80 60 40 20 ppm

9-(3-Nitrophenyl)-3,3,6,6-tetramethyl-1,2,3,4,5,6,7,8-octahydroxanthene-1,8-dione:

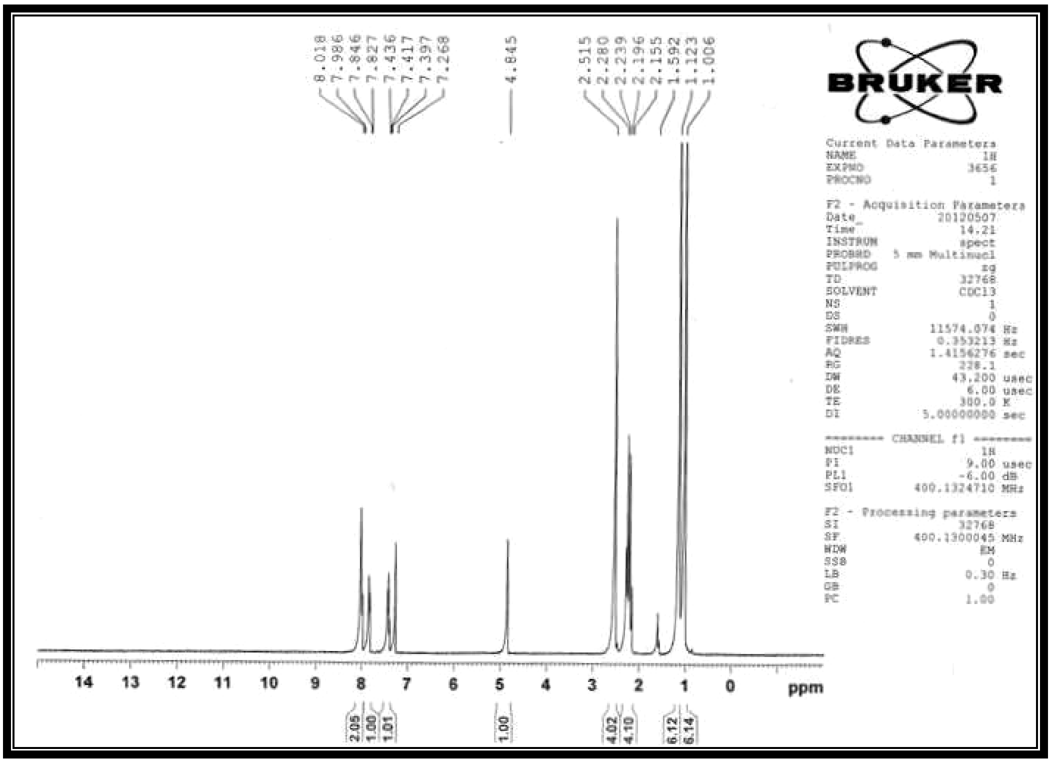

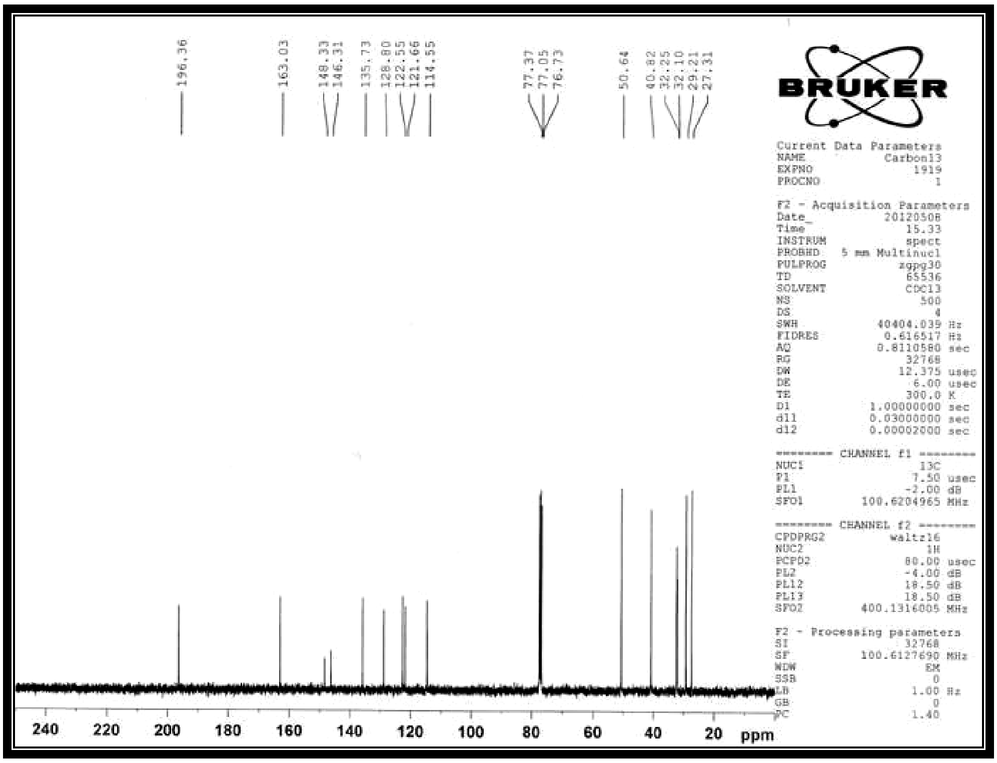

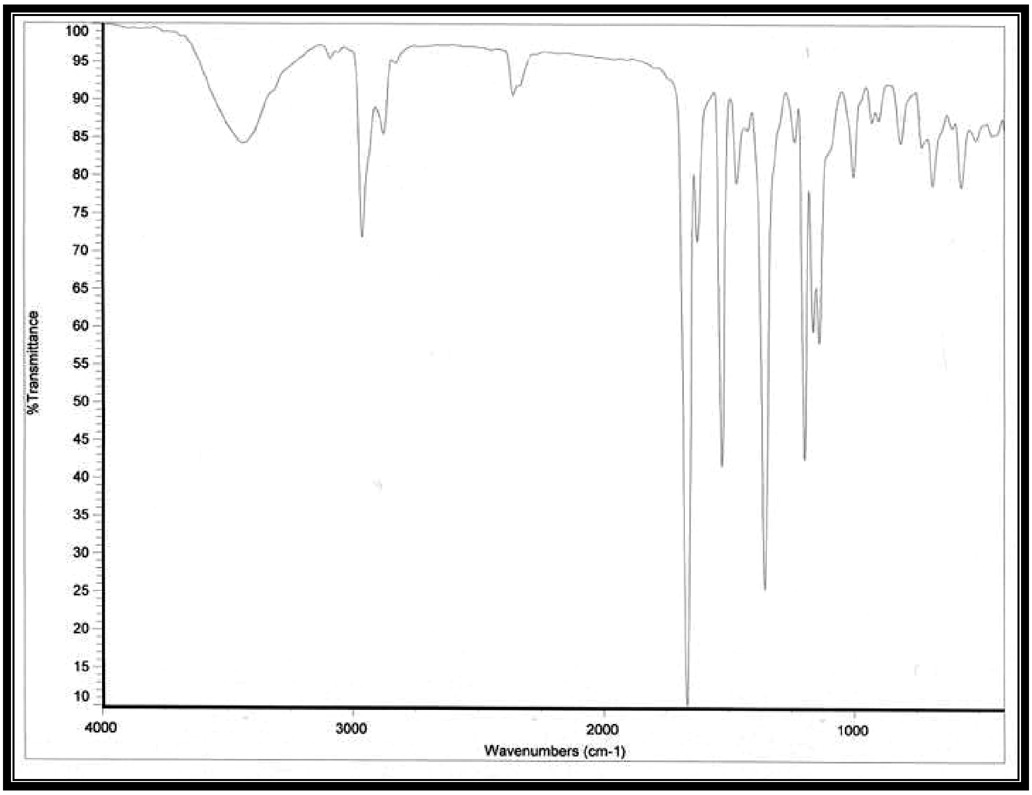
100
95
90
85
80
75
70
65
60
55
50
45
40
35
30
25
20
15
10
% Transmittance
4000
3000
2000
1000
Wavenumbers (cm-1)

9-(4-Chlorophenyl)-3,3,6,6-tetramethyl-1,2,3,4,5,6,7,8-octahydroxanthene-1,8-dione:

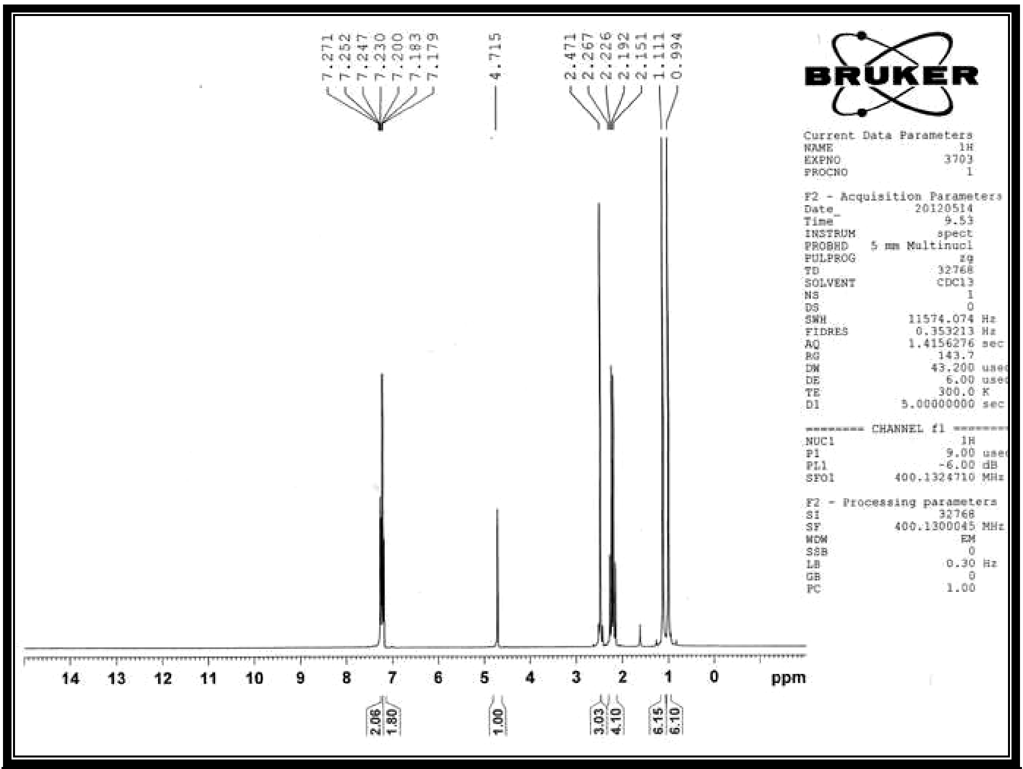

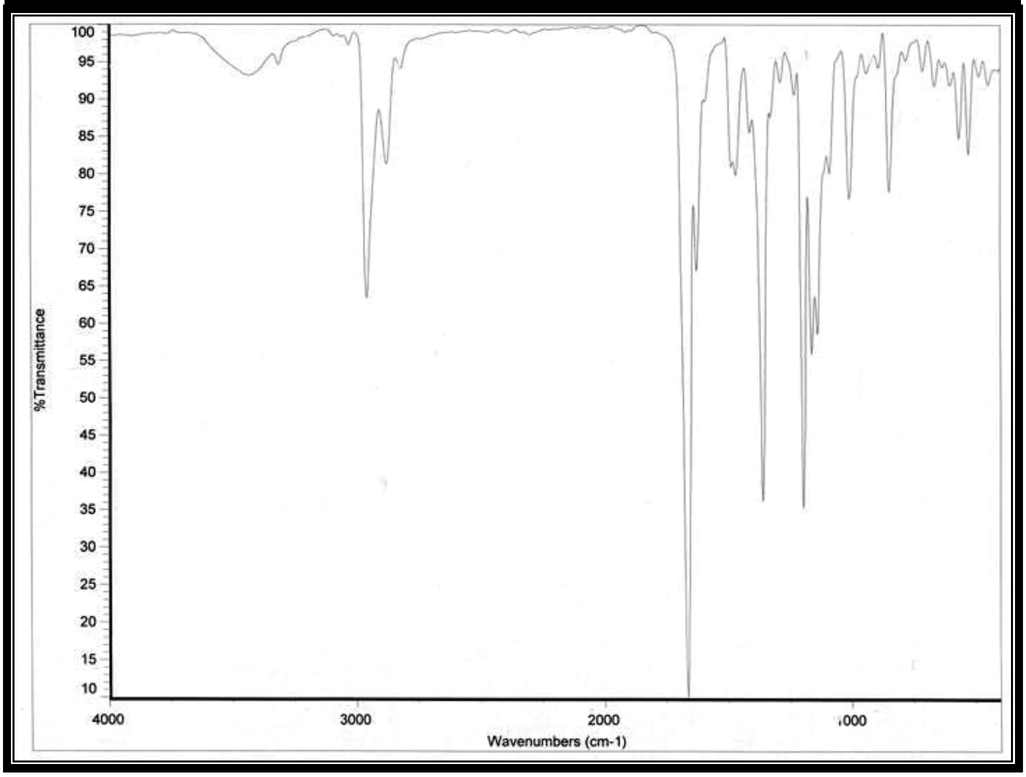

9-(4-Chloro-3-nitrophenyl)-3,3,6,6-tetramethyl-1,2,3,4,5,6,7,8-octahydroxanthene-1,8-dione:

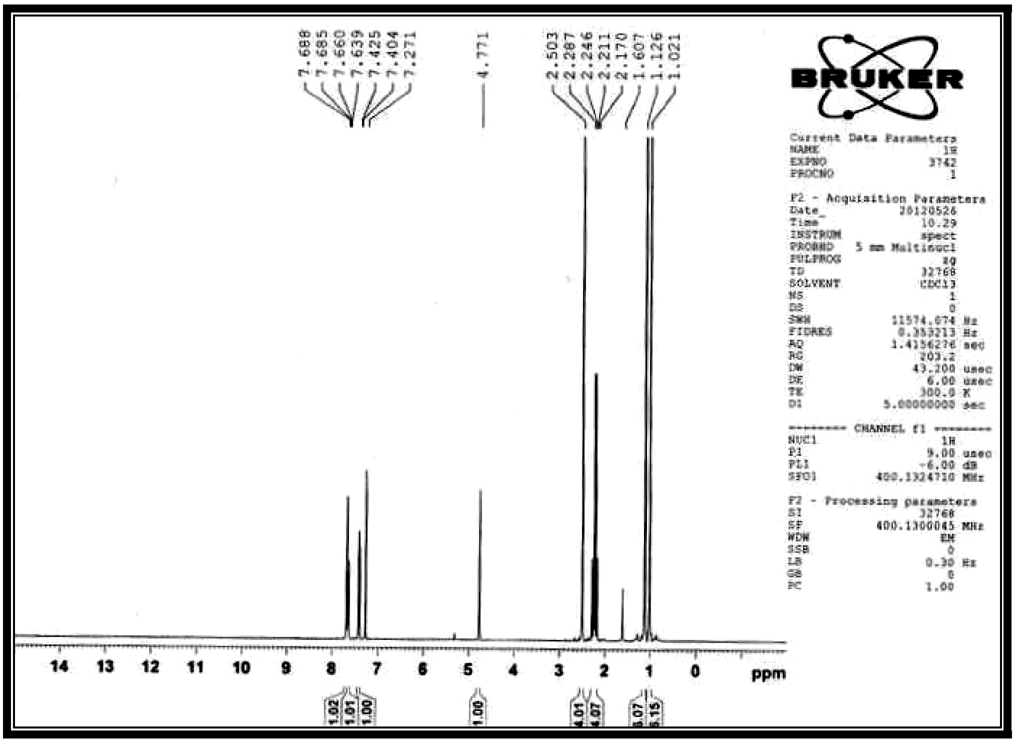

9-(2,4-Dichlorophenyl)-3,3,6,6-tetramethyl-1,2,3,4,5,6,7,8-octahydroxanthene-1,8-dione:

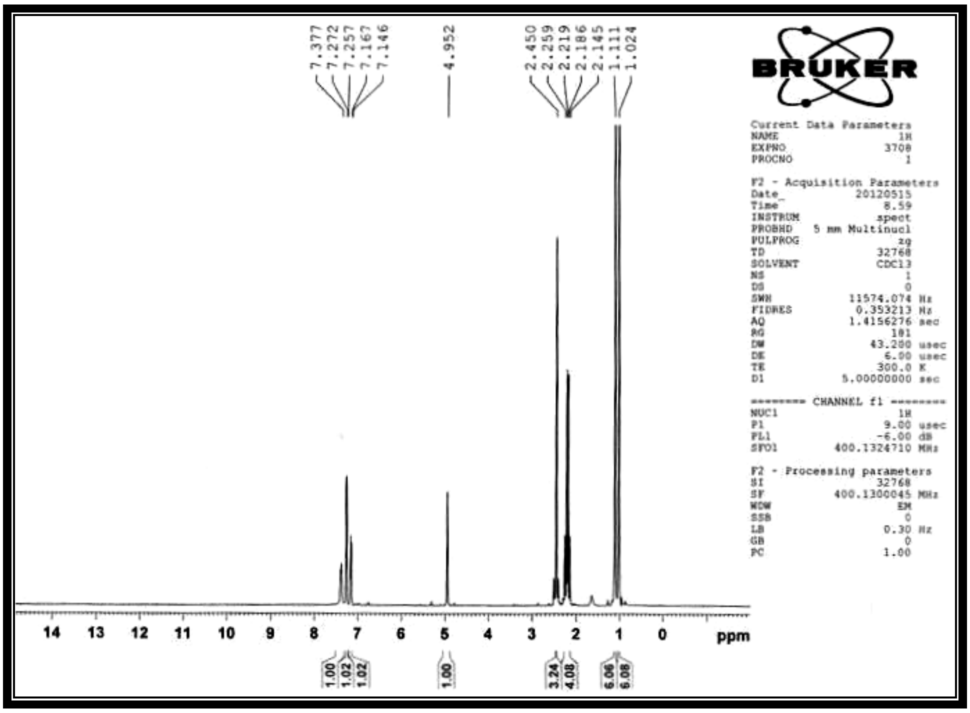

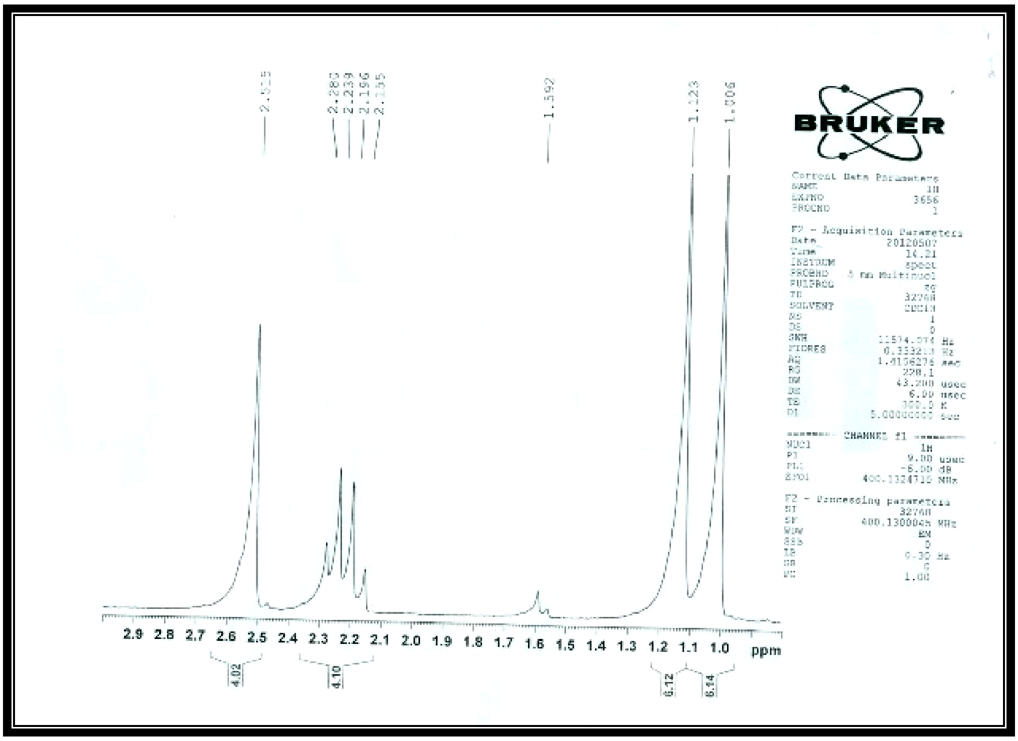

9-(3-Methoxy)-3,3,6,6-tetramethyl-1,2,3,4,5,6,7,8-octahydroxanthene-1,8-dione:

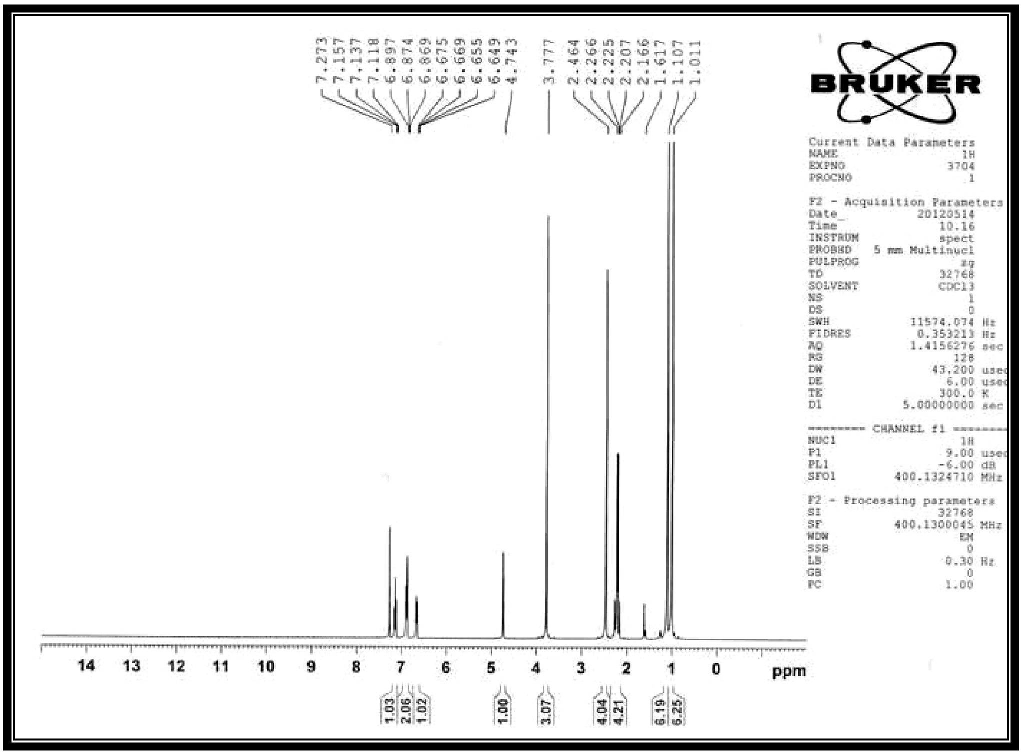

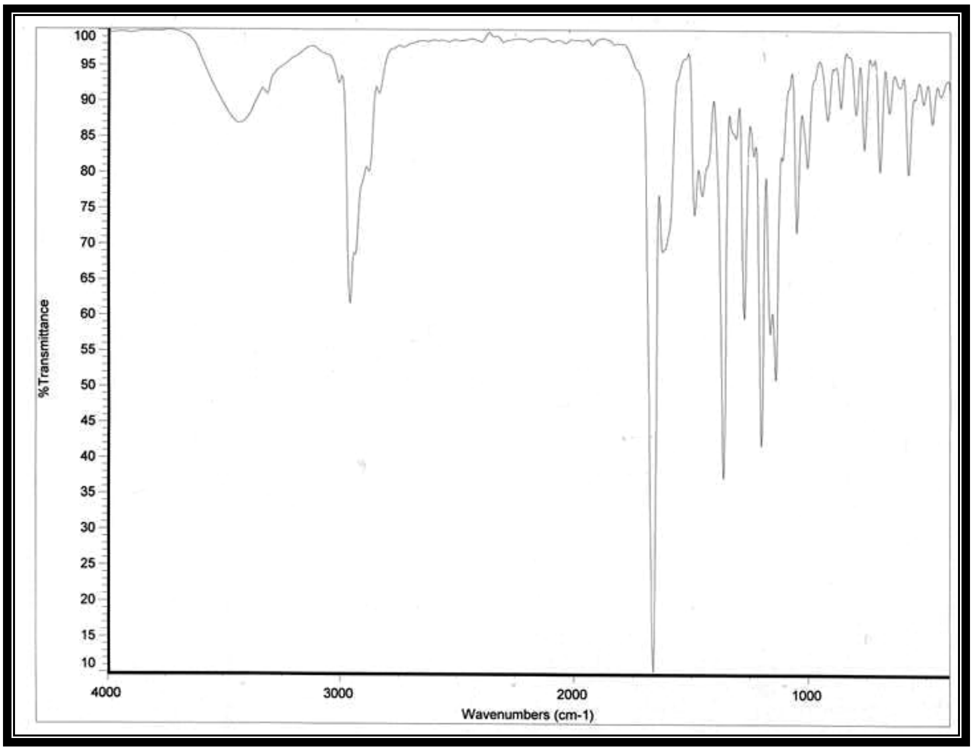

9-(4-Methoxy)-3,3,6,6-tetramethyl-1,2,3,4,5,6,7,8-octahydroxanthene-1,8-dione:

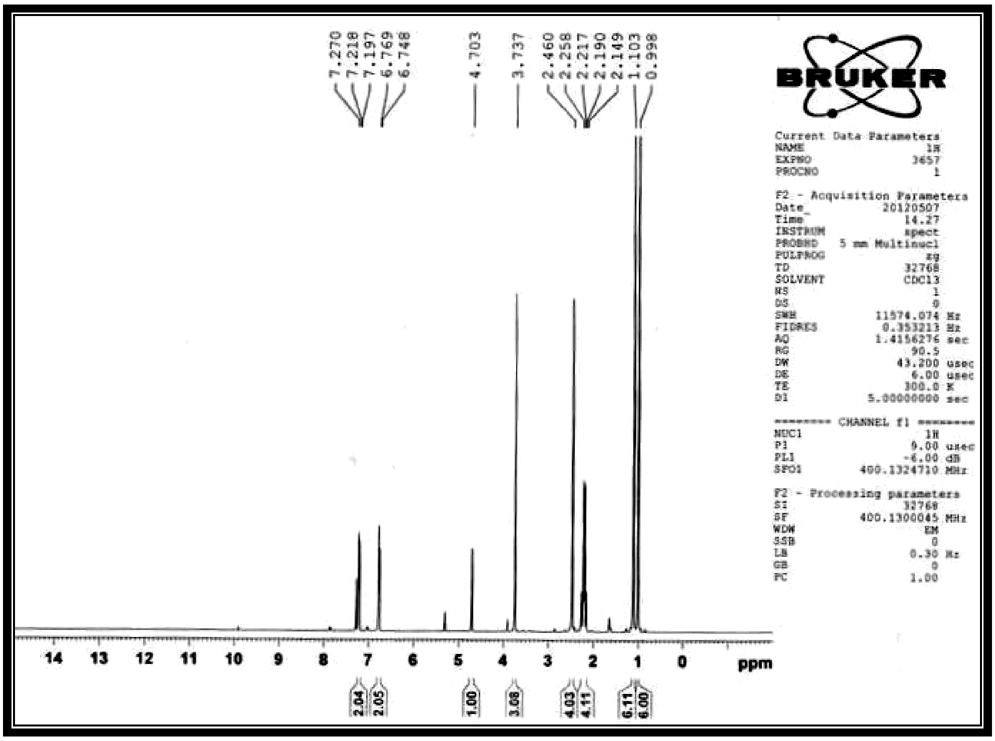

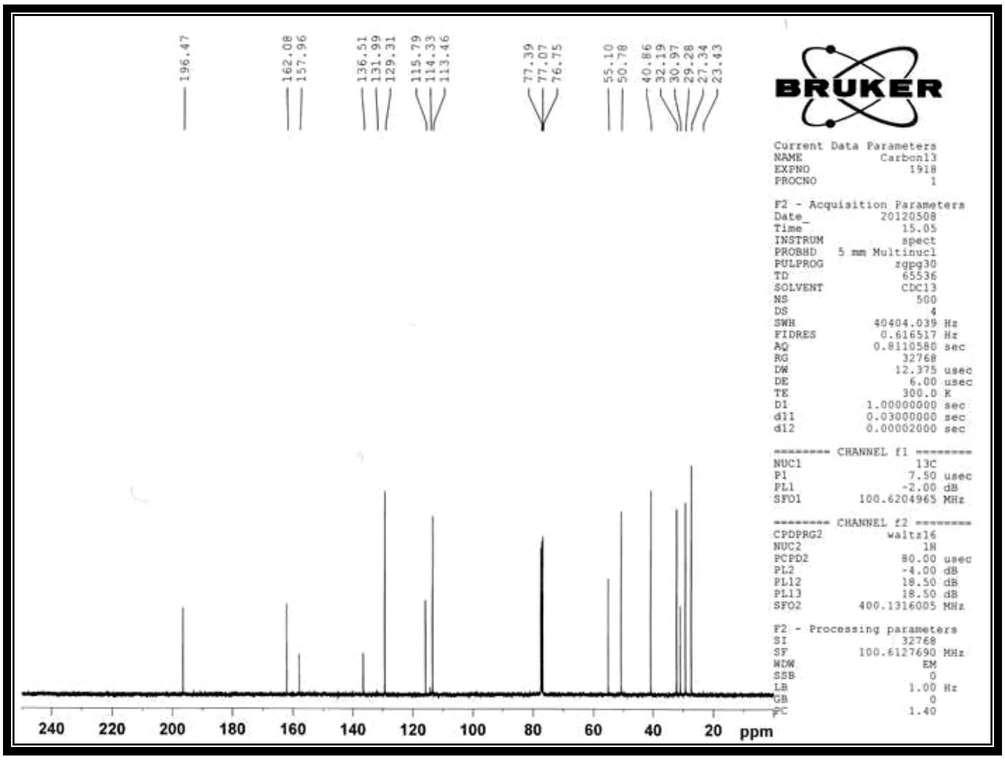
196.47
162.08
157.96
136.51
131.99
129.31
115.79
114.33
113.46
77.39
77.07
76.75
55.10
50.78
40.86
32.19
30.97
29.28
27.34
23.43
BRUKER
Current Data Parameters
NAME Carbon13
EXPNO 1918
PROCNO 1
F2 - Acquisition Parameters
Date_ 20120508
Time 15.05
INSTRUM spect
PROBHD 5 mm Multinucl
PULPROG zgpg30
TD 65536
SOLVENT CDCl3
NS 500
DS 4
SWH 40404.039 Hz
FIDRES 0.616517 Hz
AQ 0.8110580 sec
RG 32768
DW 12.375 usec
DE 6.00 usec
TE 300.0 K
D1 1.00000000 sec
d11 0.03000000 sec
d12 0.00002000 sec
======== CHANNEL f1 ========
NUC1 13C
P1 7.50 usec
PL1 -2.00 dB
SFO1 100.6204965 MHz
======== CHANNEL f2 ========
CPDPRG2 waltz16
NUC2 1H
PCPD2 80.00 usec
PL2 -4.00 dB
PL12 18.50 dB
PL13 18.50 dB
SFO2 400.1316005 MHz
F2 - Processing parameters
SI 32768
SF 100.6127690 MHz
WDW EM
SSB 0
LB 1.00 Hz
GB 0
PC 1.40
240 220 200 180 160 140 120 100 80 60 40 20 ppm

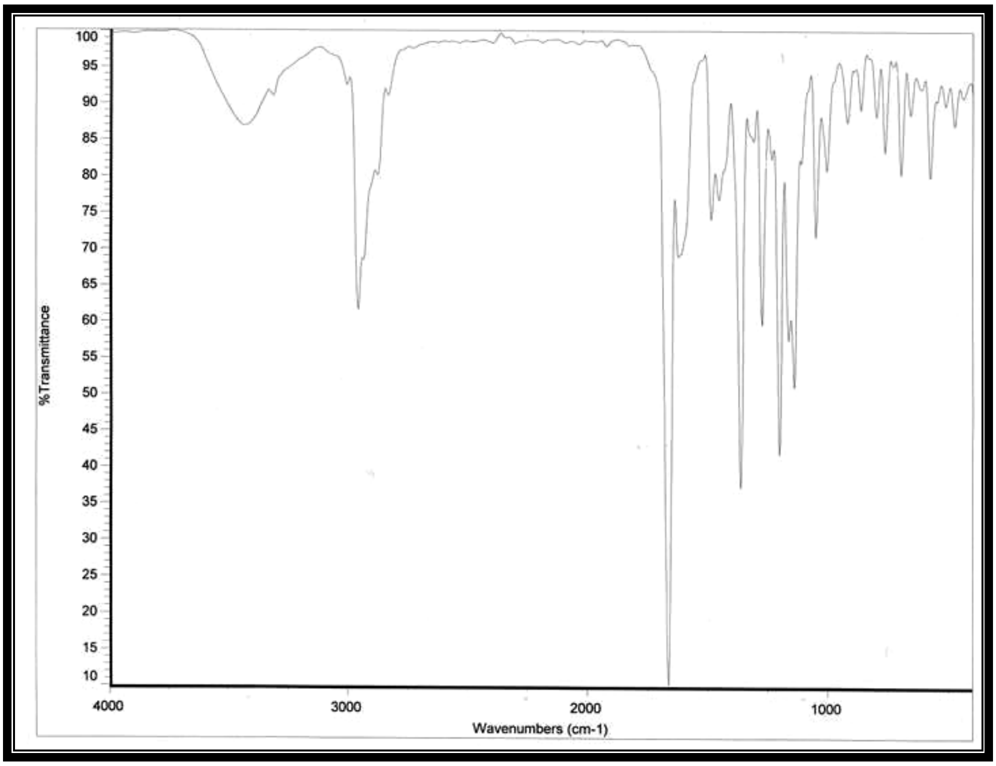
%Transmittance
100 95 90 85 80 75 70 65 60 55 50 45 40 35 30 25 20 15 10
4000 3000 2000 1000
Wavenumbers (cm-1)
